# Accumulation of Major, Minor and Trace Elements in Pine Needles (*Pinus nigra*) in Vienna (Austria)

**DOI:** 10.3390/molecules26113318

**Published:** 2021-06-01

**Authors:** Michaela Zeiner, Iva Juranović Cindrić

**Affiliations:** 1 Man-Technology-Environment Research Centre, School of Science and Technology, Örebro University, Fakultetsgatan 1, 70182 Örebro, Sweden; 2 Department of Chemistry, Faculty of Science, University of Zagreb, Horvatovac 102a, 10000 Zagreb, Croatia; ijuranovic@chem.pmf.hr

**Keywords:** metal pollution, pine needles, *Pinus nigra*, traffic volume, Vienna

## Abstract

Increasing heavy metal pollution in the environment and the fact that pine needles are good bio-monitors for air pollutants was the reason to investigate their accumulation in pine needles in Vienna (Austria). *Pinus nigra* is widespread over the city, thus allowing the study of different parameters influencing metal accumulation. The sampling sites were chosen based on traffic volume (low, medium, high). Fresh shoots were collected alongside one-year-old needles once per week from May to August 2015. The needle samples were washed and dried prior to acidic microwave-assisted digestion followed by quantitative determination using spectrometric methods. The investigation was focused on the following elements: Ag, Al, As, B, Ba, Be, Ca, Cd, Co, Cr, Cu, Fe, K, Li, Mg, Mn, Mo, Na, Ni, Pb, Se, Sr, U, V, and Zn. The one-year-old needles mainly contained higher contents of elements than fresh shoots; in many cases, the values differed statistically significantly. By correlating needle elemental contents with the sampling site, statistically significant differences were registered for the majority of the investigated elements. These differences originate from the local traffic situation, soil elemental levels, translocation processes, and not traffic-related sources. No general trend of metal accumulation from spring to summer was registered.

## 1. Introduction

In recent decades, heavy metal pollution has steadily become a serious environmental problem, because of its toxicity and insusceptibility in the environment. Since the adverse effects to the environment and beings are more important for the characterization of an element than its density, the term potentially toxic elements (PTE) has been suggested instead [1]. Due to accumulation in crops or plants, these elements may harmfully affect animal or human physiological functions through the food chain [2,3,4]. Especially cadmium and lead are considered elements with adverse effects on animal and human health as they are readily transferred through food chains and are not known to serve any essential biological function [5]. Furthermore, the increasing number of automobiles has been found to be an influencing parameter for the high concentration of lead, platinum, and palladium in the environment (air and soil), not only in urban zones but also in remote areas [6,7].

Vegetation is considered a valuable indicator of environmental contamination by PTE via root uptake on the one hand and via precipitation on the outer surface, e.g., leaves and bark, on the other hand. Thus, perennial plants especially can be used to monitor pollution across both spatial and temporal scales. Of prime importance for the study of trace element pollution specifically in remote areas is the choice of suitable accumulative bio-monitors. Pine needles represent good bio-monitors of air pollutants, due to their waxy surfaces, which are prone to accumulate gaseous pollutants as well as polluting particulates [8,9].

Regarding metal pollution, pine needles have been studied in urban and remote areas in different parts of the world during the last decades. Al-Alawi and coworker investigated Aleppo pine needles (*Pinus halepensis*) in Amman City, Jordan, for their contents of Cd, Cu, Pb, and Zn [10]. A Spanish working group determined Sb, Cd, Pb, Ni, Cu, Cr, Ti, Zn and Al in needles of the same pine species collected in Barcelona and surroundings [11]. Yilmaz and colleagues focused on the determination of Cu, Pb and Zn in pine needles in Turkey [12]. A Chinese group investigated the influence of industrial activity on the contents of Cu, Cd, Pb, Zn, Cr, Ni, and Co in Masson pine needles (*Pinus massoniana*) [13], stating a correlation of higher needle metal amounts and industrial regions. Furthermore, traffic has been found to influence the elemental pattern of needles [14]. Sawidis and colleagues stated that the kind and intensity of pollution determines the final metal content in the needle material. Their conclusion is built on the analysis of Cr, Cu, Fe, and Pb in black pine needles (*Pinus nigra*) from three different European cities [15]. Another study on differences in metal needle contents given by the geographical origin is based on Aleppo pine needles along the Croatian coastside [16]. The fact that the metal uptake behavior of plants depends on the species has been shown not only for deciduous trees [17,18] but also for conifers. The elemental pattern determined in needles from the arboretum of the University of Zagreb revealed significant differences between various pine species [19]. Hybridization has been proven to may alter this behavior [20]. Using perennial needles as bio-monitors offers the possibility for long-term studies. Polluting metals tend to accumulate over time [21], whilst the contents of others, mainly essential elements, decrease with needle age [22]. This behavior is determined by the mobility of the respective element in the plant [22].

The present investigation was designed based on the characteristics and findings of the above-listed publications. Only one representative pine species was selected for sampling in order to exclude species-dependent influences. Three locations within one city covering different traffic volumes were chosen to see the impact of pollution. Fresh shoots and one-year-old needles were collected to see the accumulation over time. Seasonal changes were supposed to be investigated by sampling in spring and summer. Since the majority of published articles deal only with (local) pollutants, the range of analytes was widened including also essential elements [12,13,14,15].

*Pinus nigra* J.F. Arnold, also called Austrian pine or black pine, is a characteristic plant of the eastern Alps boundary. It was first described botanically in Austria in 1785 [23,24]. The light- to dark-green needles can reach a length of up to 20 cm and an age of three to eight years. Due to its high amount of resin, the tree became economically important for pine tapping, mainly until the end of the 1970s. Recently, an organic chewing gum based on resin was brought onto the market [24]. Apart from the widespread natural occurrence in eastern Austria, this tree was also used in course of afforestation within the urban area of Vienna [25]. Nowadays, black pines are found all over the city; thus, it can be considered as a representative species in the chosen city.

Summarizing, the objective of the present study was the quantitative determination of twenty-five elements (metals and metalloids) in pine needles collected at different sites in the Austrian capital Vienna during one vegetation period. This sampling strategy ensured a focus on potential variances in time and location to investigate different influences of traffic volume. Collecting fresh shoots (grown in the year of sampling) as well as one-year-old needles (shoots from the year before sampling) offered the additional possibility to see differences in the elemental accumulation depending on needle age. The elements chosen include essential and harmful ones, as well as some without known physiological function in the plant.

## 2. Results

### 2.1. Analytical Methodology

In the course of method validation in previous studies [17,20], the instrumental conditions for both instruments have already been tested and found to be applicable for the given analytical task. All calibration curves had R^2^ values higher than 0.999. Since a slightly different acid digestion method for sample preparation was used in this investigation, common figures of merit for the analytical procedure applied were determined (re-validation). The calculated limits of quantification (LOQs) for all analytes were below 2 mg/kg of dried needle material. Analysis of the standard reference material for pine needles (SRM 1575a) led to recoveries ranging from 91% to 107% for the certified elements, thus the trueness of the results is proven. The precision was determined up to 2.7%. The day-to-day repeatability for all analytes was <2.9%. Good trueness and precision showed satisfying accuracy of the applied procedure. The obtained values (listed in Table S1) are similar to those from previous studies [16,19,20]. In addition, the needles were carefully washed after sampling to avoid interference by deposited material containing the elements of interest.

### 2.2. Metals and Metalloids in Pine Needles

**Figure 1:**
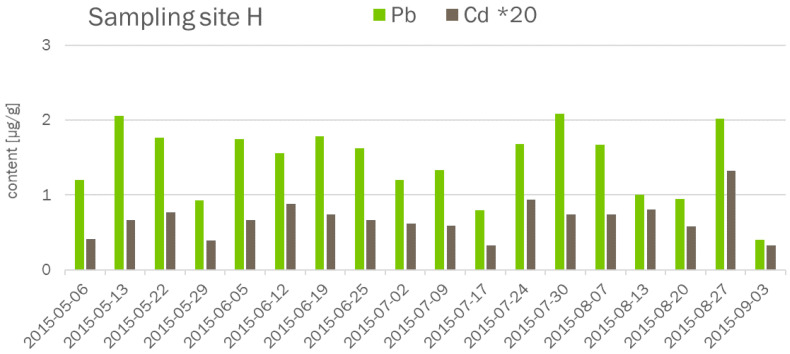
Contents of Cd and Pb in needles collected at sampling site H for the entire sampling period.

Twenty-five elements, namely Ag, Al, As, B, Ba, Be, Ca, Cd, Co, Cr, Cu, Fe, K, Li, Mg, Mn, Mo, Na, Ni, Pb, Se, Sr, U, V, and Zn, were quantitatively determined in needles of black pines at different sampling sites during one vegetation period. In order to see how to group the results, the first step of data evaluation covered the potential time trend in element accumulation in the one-year-old needles from all sampling sites during the sampling period from spring to summer. To illustrate the temporal variances in metal content, the data for Cd and Pb in the samples from site H are plotted in Figure 1.

**Figure 2:**
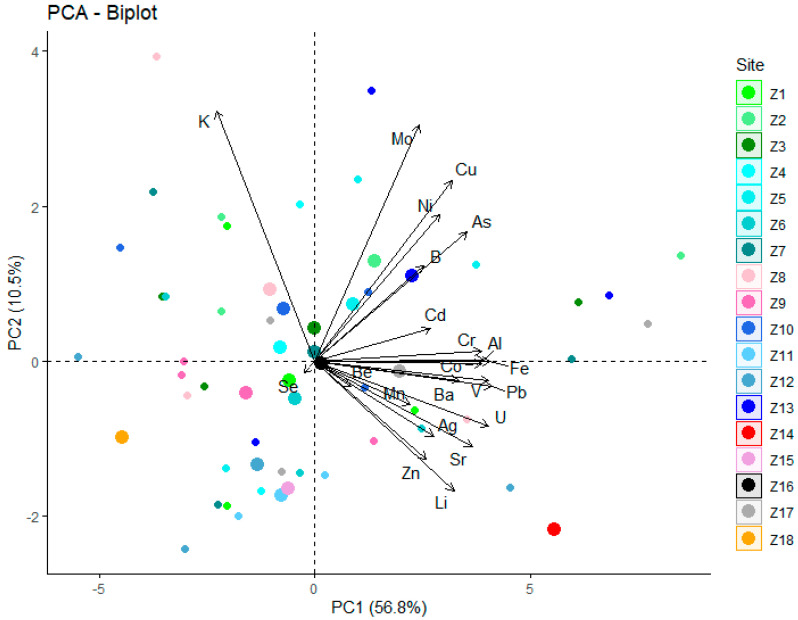
PCA biplot - sampling time, whereby Z1 to Z18 stand for the 18 weeks of sampling.

In addition, principal component analysis based on the sampling time showed no statistically significant tendency of accumulation of one the investigated elements, as can be seen in Figure 2.

Since, based on the statistical evaluation shown above, no significant tendency in time was found for all elements analyzed, all data for one-year-old needles from each sampling site were merged and further treated as one data set. A summary of these values including minimum and maximum alongside median is presented in Table 1.

## 3. Discussion

Based on the Neumann’s trend test (level of significance 95%), there was no statistically significant trend in elemental content for any of the analytes at each of the three sampling sites, which was also proven by PCA. These findings correspond to results published by other researchers. A Turkish working group determined Cu and Co levels in *P. nigra* needles from six different samplings sites four times a year to test for seasonal changes [26]. Their results show variance over the year, but no statistical test was performed to check for statistically significant changes. This behavior is similar to perennial grass species, such as *Phragmites australis* (common reed), where increasing contents in the leaves were found for Cd, Co, Cr, Cu, Mn, Ni, Pb, Zn, Sr, and V during and after the growing season until August and October, respectively [27]. This accumulation tendency has also been reported for six metals (Cd, Cr, Cu, Ni, Pb, and Zn) in leaves of deciduous trees, namely willow species, by Dutch researchers [28]. Apart from this seasonal trend, the metal uptake differs between herbs and trees, in both cases being additionally species-dependent [29,30].

Needle contents of various metals and metalloids have been studied by different research groups. Comparing the results is not an easy task, since the needle contents depend on many different factors, e.g., geographical location [15]. Not only the place, determined by the exposure via air and soil, i.e., the actual environmental pollution or background levels, but also the climatic conditions influence the element accumulation [16]. Furthermore, the year of sampling affects the obtained results for needles sampled in the same area [30]. Needles collected in Cologne (Germany) in 2004 were reported to have element contents in similar ranges as those obtained in the present study regarding Ba, Ca, Cd, Fe, Mo, Na, and Pb, i.e., for major, minor, and trace elements [14]. A comparative study of metals in *P. nigra* needles from three different European cities, i.e., Salzburg (Austria), Belgrade (Serbia), and Thessaloniki (Greece), focused on four metals considered to be environmental pollutants, namely Cr, Cu, Fe, and Pb [15]. Whilst their values for Cu and Fe are in the same range as those from Vienna, the Cr and especially the Pb contents found by Sawidis and colleagues are much higher [15]. Belgrade was also the sampling site of another investigation, which quantified Fe, Mn, Ni, Pb, and Zn in black pine needles [30]. Whereas the needles from the Viennese trees contain less Ni, the Serbian results are higher regarding Ni. The results for Pb, Zn, and Mn (except for one location) are in the same range. Black pines growing in Gaziantep and surroundings (Turkey) have lower Cr needle contents but higher Ni needle contents compared to the black pines from Vienna [31]. Besides these papers dealing with only a few elements, needles from different pine trees grown in the arboretum of the University of Zagreb have been analyzed for 21 elements [19]. The results reported for *P. nigra* needles show lower contents for Al, B, Ba, Ca, Cr, Fe, K, Li, Sr, and Zn and higher contents for Mo, Na, Pb, and Se, while the levels of As, Cd, Co, Cu, Mg, Mn, and Ni are in a similar range. The importance of the sampling site as an influencing factor for metal accumulation is underlined by all these studies. Not only does the anthropological impact on a certain place determine elemental contents in soil, but also the natural background is of importance; their ratio not always being easy to be determined. Many metals and metalloids, such as As, Cd, Co, Cu, Cr, Ni, Sr, U, and Zn, are naturally occurring in soil and environment, and their levels being determined by weathering processes; thus, they may be transferred to plants and water [32].

**Figure 3:**
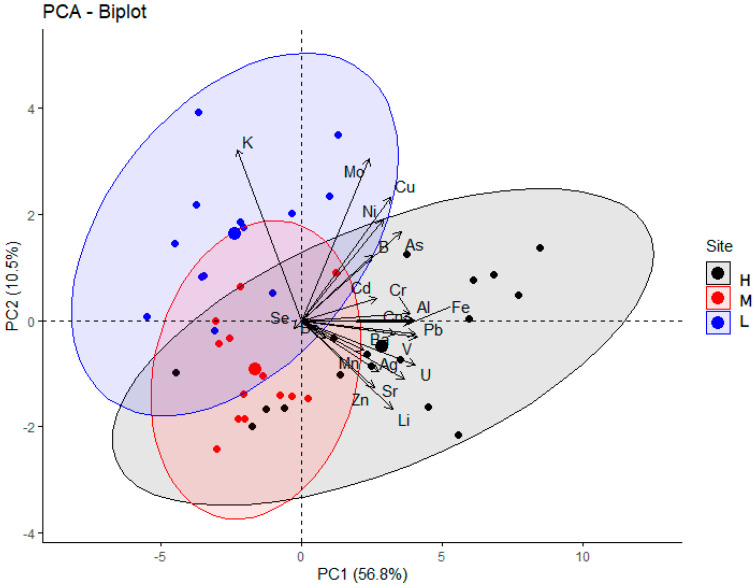
PCA biplot—sampling sites.

**Figure 4:**
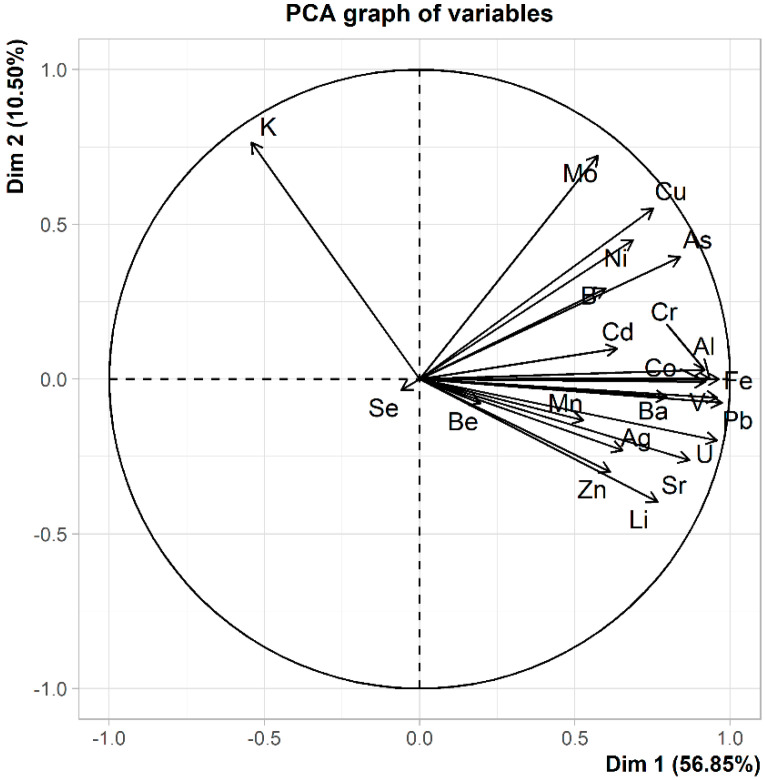
PCA plot—contributions of variables (elements investigated).

Regarding the sampling site and thus the traffic volume the elemental needle contents were tested for statistically significant differences by different methods. On the one hand, the entire data set of all elements in each site were analyzed by ANOVA (*p* > 0.2) and PCA, whilst on the other hand, the needle contents were compared for each element using paired *t*-tests; the results of the latter are given in Table 2. While no statistically significant difference can be seen, when focusing on all elements determined (see Figure 3), fifteen of the investigated elements were found to differ statistically significantly between the sampling sites (see Table 2); their contributions are shown in Figure 4.

These are As, B, Ba, Be, Cd, Cr, Cu, Mo, Na, and Se. Boron, for example, is an element naturally occurring in the environment due to the release into air, soil, and water through weathering. It is less impacted by manufacturing glass, combusting coal, melting copper, and through the addition of agricultural fertilizers [33]. Furthermore, the results for Ag, Ni, and Fe can be considered more or less site-independent. The former two are only statistically significantly different between the places M and H, with the latter only between L and H.

**Figure 5:**
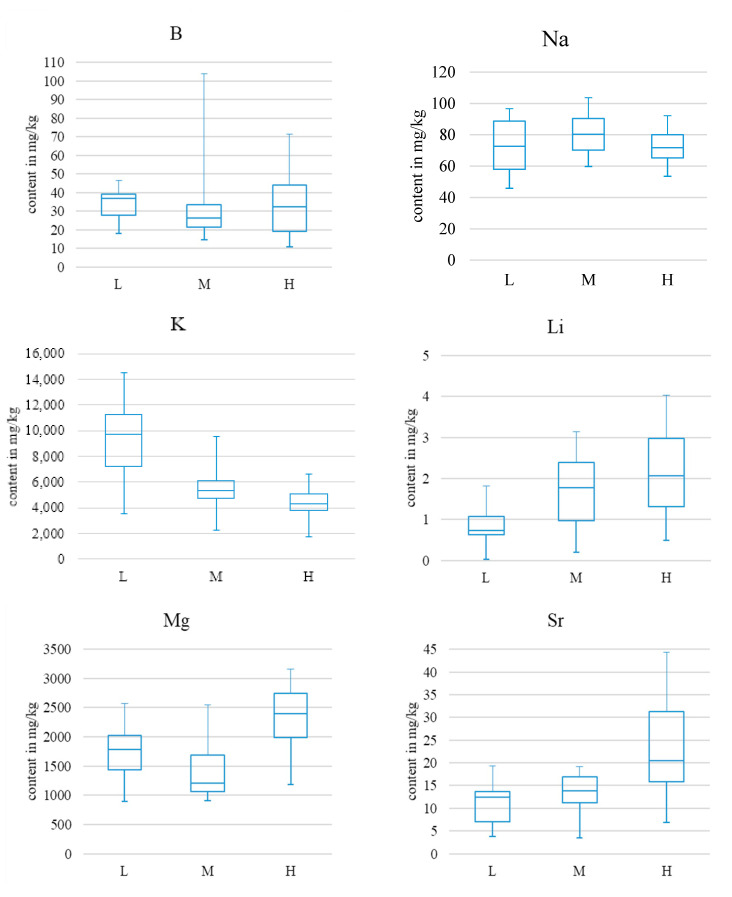

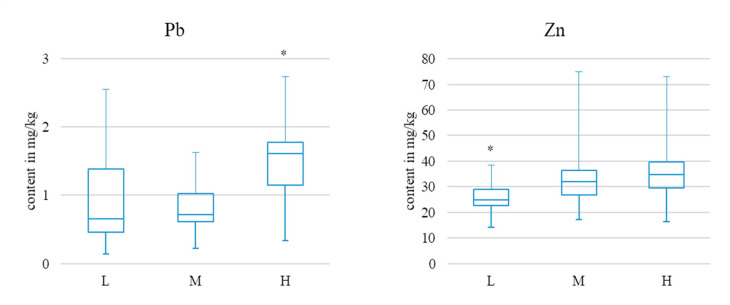
Box-and-whisker plots for selected elements Ba, Na as examples for notbeing statistically different; K, Li, Mg, and Sr being different between all sampling sites; Pb and Zn being different at one site. (L = low traffic volume; M = medium traffic volume; H = high traffic volume). *—statistically significant difference.

Conversely, the needle contents of K, Li, Mg, and Sr differ statistically significantly between all sampling sites (see Figure 5). Lithium is used in batteries, ceramics, air-conditioning, grease, and electric cars and is considered an emerging contaminant. In particular, the introduction of the lithium-ion battery technique led to a significant increase in its production during the last century [34]. Thus, changes in its environmental distribution are to be expected, e.g., through the disposal of lithium-containing products. While geogenic Li is sparingly soluble, plants easily take up ionic Li [35]. As for Sr, the Li needle contents rise with traffic volume. A converse trend is seen for the plant nutrient K, displaying a decreasing trend. The Mg contents do not follow the order of traffic density, but H > L > M.

In the needles sampled at high-traffic sites, the contents of seven elements, namely Al, Ca, Co, Mn, Pb, U, and V, are statistically significantly higher than in those from the other two sampling sites. Conversely, only one investigated analyte (i.e., Zn) displays the statistically significant lowest needle content at sampling site L (low traffic place). The box-and-whisker plots for Pb and Zn are given in Figure 5 as representative elements for being different at one site. Lead levels in the environment are found to decrease after phasing out leaded gasoline [36]. The high Pb contents at site L are a result of high Pb content in soil resulting from a local source.

Analysis of the data obtained for the fresh shoots compared to the one-year-old needles revealed that the contents of Ag, (Al), B, Ba, Co, Cr, Fe, K, Li, Ni, Pb, Se, Sr, U, and V differ statistically significantly (see Table 3).

Except for the plant nutrients K and Ni, higher amounts were found in the older needles. These results are in agreement with literature data of various conifer species. Similar findings were published already twenty years ago, whereby even Cu was found to decrease in the black pine needles studied in the 1990s [22]. Longer exposure time usually results in higher foliar element contents [14]. The increasing trend of metal contents in perennial needles, even for Ni, was also found in a more recent study by a Turkish research group who investigated 1-, 2-, and 3-year-old needles collected from *Pinus sylvestris*, *Pinus nigra*, *Abies bornmülleriana*, and *Picea pungens* [21]. Different organs of blue spruce (*Picea pungens* Engelm), namely needles, bark, and branches, also show statistically significant increases in Mn content with age in the range from one to seven years [37].

## 4. Materials and Methods

**Figure 6:**
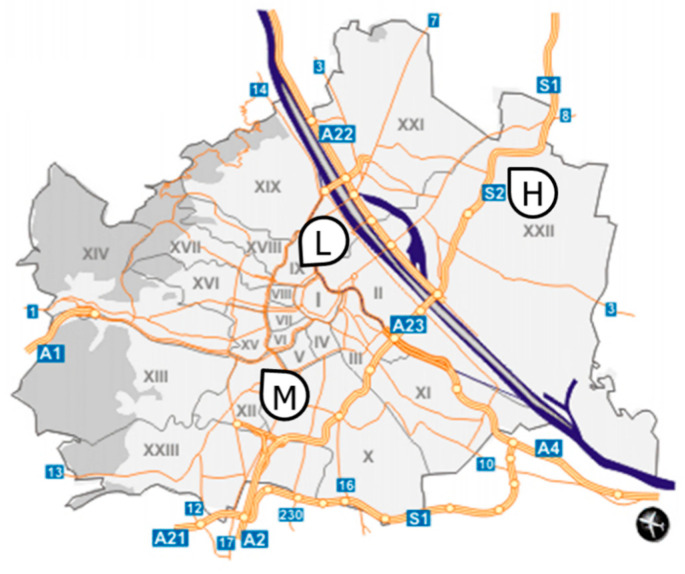
Map of Vienna [40] with sampling sites (L—low traffic volume; M—medium traffic volume; H—high traffic volume).

Fully developed one-year-old pine needles from *P. nigra* trees were collected weekly from May to August 2015 in Vienna at three sites characterized by different traffic volumes (see Figure 6). No traffic count at the roads at sampling sites is available; thus, data from the vicinity were taken for assessment. Traffic counts are performed on municipal roads classified as main roads categories A and B every 5 years [38] and on highways monthly [39]. The data for H can be estimated by the daily number of vehicles on a municipal road at 1.5 km linear distance from the sampling site with ~17,000 [38] and that of vehicles on the highway counted at approx. 4 km linear distance from the sampling site monthly, in May 2015 being ~43,000, in June 2015 ~46,000, in July 2015 ~50,000, and in August 2015 ~49,000 [39]. Sampling site M is located close to a crossing of a short minor one-way road with only one lane and no permanent through traffic and a two-way road (two lanes). The traffic volume can be estimated to range from 8000 to 10,000 vehicles per day based on a vehicle counting point on a parallel road of the former (separated by one block of houses) [38]. Sampling site L is situated in a park without regular traffic permitted.

The selected sampling sites were not pine forests but places where approximately 10 to 20 black pines grow. Trees of similar age (based on trunk similar trunk circumference) were chosen at each sampling site, and the number of trees was 6 for sampling site L and 8 for M as well as H. In addition, fresh shoots were sampled once per week in July 2015 from the same trees. Needles and shoots were collected from approximately the same height and the crown site facing the road. The needles from each tree and sampling time were stored in PE bags and further treated as pooled sample. After sampling, the needles were stored in a refrigerator prior to sample preparation. Once per month A-horizon soil samples were collected at each of the three sampling sites, their pH values ranging from 5 to 7.

In order to quantify the amounts of the elements of interest resulting in the needles by precipitation, the needles were rinsed with triple distilled water (in-house production). For the determination of the taken-up amount of the elements investigated, the washed needles were dried at 105 °C, milled using a metal-free device, weighed, and digested. This wet digestion step was performed using a closed-vessel microwave-assisted digestion system (MLS-1200 MEGA) using a mixture of 5 mL subboiled nitric acid (HNO_3_; Merck, Darmstadt, Germany) and 1 mL hydrogen peroxide (H_2_O_2_; Merck, Darmstadt, Germany; 30% *w*/*w*). The following temperature program was applied (time in min/power in W): 3/250; 1/0; 4.5/250; 6/650; 5/400; ventilation 25 min. The final volume of 10.0 mL was achieved by adding ultrapure water. Repeated digestion was done for each sample, based on 100 mg to 150 mg aliquots each. Reagent blank solutions were prepared in the same way. The blank solutions were measured ten times, and the standard deivation (*s*) was used to calculate the limits of detection (LODs) and limits of quantification, based on 3 *s* and 9 *s*, respectively.

For quality assurance, the standard reference material SRM1575a—Trace Elements in Pine Needles (NIST, Gaithersburg, ML, USA)—was processed as described for the sample needles. The validity of the applied analytical procedure was checked by determining the recoveries and the precision alongside the day-to-day repeatability for the analytes. In order to minimize potential contamination, all glass- and plasticware was pre-cleaned with semi-concentrated nitric acid prior to use.

Prior to measurements by inductively coupled plasma sector field mass spectrometry (ICP-SFMS; Thermo Elemental Corporation, Finnigan Element 2; Bremen, Germany), a further dilution step 1:20 was carried out. ICP-MS was used to quantify Ag, Al, As, B, Ba, Be, Cd, Co, Cr, Cu, Fe, Li, Mn, Mo, Ni, Pb, Se, Sr, U, V, and Zn. In addition, the digest solutions were analyzed by inductively coupled plasma optical emission spectrometry (ICP-OES; Prodigy HD; Teledyne Leeman, Hudson, NH, USA) for the major elements Ca, K, Mg, and Na. Both analytical methods have been optimized before [16], and the operational conditions are listed in Table 4. The quantification was performed via external calibrations based on multi-elemental standards solutions. Respective dilutions were prepared using 2.5% *w*/*w* nitric acid and ICP Multielement Standard IV (Merck, Darmstadt, Germany) as a stock solution (1000 mg/L).

Data were evaluated considering reagent blank, final volume, dilution step, and mass of sample digested so that the final contents were obtained in mg/kg of all analytes for each dried needle sample. Paired *t*-tests were applied to check for statistically significant differences between needles of different ages. Calculation of the significance of the difference in elemental needle content between the sampling sites was performed for all elements and places individually using paired *t*-tests and as entire data sets using one-way analyses of variance (ANOVA). The decision for a significant time trend during the vegetation period was based on Neumann’s trend test. Furthermore, Principal Component Analysis (PCA) was carried to determine the influence of the sampling site and sampling time and the contribution of each element analyzed. All calculations were carried out using Microsoft Office Excel, versions 2013 and 2016 or R 4.03. A level of significance of 95% was used for decision-making in all statistical tests performed.

## 5. Conclusions

The findings of the presented study on metals and metalloids in pine needles did not show a general trend of metal accumulation from spring to summer. The influence of needle age on the metal accumulation could be proven by the fact that older needles mainly contained higher contents of elements, and in many cases, their contents differed even statistically significantly. Furthermore, the correlation of the needle elemental contents with the different sampling sites revealed statistically significant differences for most of the investigated elements. Taking a closer look at the single elements, it can be clearly seen that these differences not only originate from the local traffic situation, i.e., airborne pollution and uptake via the needles, but are also based on soil elemental levels, translocation processes, and not traffic-related sources.

## Figures and Tables

**Table 1:** Elemental content in the dried needle samples from all sampling sites in mg/kg.

Element	Sampling Site L			Sampling Site M			Sampling Site H		
	Min	Max	Median	Min	Max	Median	Min	Max	Median
Ag	<LOQ	0.0272	0.0084	<LOQ	0.0170	0.0079	<LOQ	0.0254	0.0136
Al	38.1	380	105	51.5	193	110	61.0	490	230
As	<LOQ	0.249	0.121	<LOQ	0.538	0.080	<LOQ	0.360	0.152
B	17.8	46.6	37.0	14.7	104	26.1	10.7	71.3	32.5
Ba	0.982	10.6	4.37	2.17	14.0	5.62	1.60	10.7	6.08
Be	<LOQ	0.0197	0.0101	<LOQ	0.0238	0.0084	<LOQ	0.0192	0.0063
Ca	1849	11658	6325	1534	9027	6292	3159	14735	8024
Cd	0.0044	0.0843	0.0244	0.0080	0.0807	0.0259	0.0084	0.0662	0.0334
Co	0.038	0.183	0.074	0.035	0.180	0.068	0.036	0.294	0.131
Cr	0.056	1.96	0.668	0.441	4.52	0.754	0.236	2.83	1.15
Cu	3.09	12.2	5.42	3.07	25.1	4.87	2.49	15.9	5.72
Fe	36.9	581	177	112	993	240	92.4	809	381
K	3547	14511	9697	2230	9512	5294	1690	6619	4275
Li	0.026	1.82	0.736	0.199	3.15	1.78	0.490	4.03	2.07
Mg	902	2571	1787	909	2542	1203	1185	3160	2334
Mn	10.3	77.7	26.5	13.4	92.3	29.4	12.1	117	46.4
Mo	0.176	0.866	0.343	0.119	1.79	0.351	0.145	1.23	0.290
Na	45.8	96.7	72.7	59.8	104	80.0	53.5	92.2	71.5
Ni	0.186	1.01	0.579	0.251	1.14	0.410	0.226	1.18	0.550
Pb	0.137	2.55	0.655	0.228	1.63	0.714	0.333	2.74	1.61
Se	0.019	0.295	0.096	<LOQ	0.488	0.051	<LOQ	0.334	0.094
Sr	3.85	19.3	12.4	3.47	19.1	13.8	6.82	44.3	20.5
U	<LOQ	0.027	0.0085	<LOQ	0.038	0.012	<LOQ	0.036	0.024
V	<LOQ	0.921	0.208	0.067	0.688	0.260	0.114	0.887	0.498
Zn	14.2	38.5	24.8	17.1	75.1	32.0	16.4	72.9	34.7

**Table 2:** Statistically significant differences in elemental content in the dried needle samples between the sampling sites when the respective site combination is marked with *.

Element	Statistically Significant Difference		
	L-M	L-H	M-H
Ag			*
Al		*	*
As			
B			
Ba			
Be			
Ca		*	*
Cd			
Co		*	*
Cr			
Cu			
Fe		*	
K	*	*	*
Li	*	*	*
Mg	*	*	*
Mn		*	*
Mo			
Na			
Ni			*
Pb		*	*
Se			
Sr	*	*	*
U		*	*
V		*	*
Zn	*	*	

**Table 3:** Mean elemental contents in mg/kg (elements with statistically significant differences between fresh shoots and one-year-old needles (1a) are written in italic) alongside trend (**↑**—increasing; ↓—decreasing).

Element	Fresh Shoots	1a	*p*-Value	Trend
Ag	0.0022	0.013	0.014	↑
Al	<LOQ	116		↑
As	0.053	0.102	0.098	(↑)
B	18.3	30.4	0.005	↑
Ba	1.35	4.94	0.00033	↑
Be	<LOQ	0.00159		↑
Ca	2268	7396	0.00049	↑
Cd	0.033	0.036	0.635	
Co	0.058	0.082	0.033	↑
Cr	0.102	0.727	0.001	↑
Cu	6.04	4.83	0.081	(↓)
Fe	35.9	214	0.001	↑
K	11577	7039	0.00018	↓
Li	0.129	1.364	0.002	↑
Mg	1466	1975	0.056	(↑)
Mn	27.7	38.5	0.181	(↑)
Mo	0.407	0.275	0.193	(↓)
Na	59.1	67.9	0.178	(↑)
Ni	0.743	0.406	0.005	↓
Pb	0.162	0.912	0.003	↑
Se	0.026	0.079	0.010	↑
Sr	6.23	19.3	0.006	↑
U	0.0018	0.0121	0.004	↑
V	0.027	0.268	0.002	↑
Zn	32.3	28.8	0.436	(↓)

**Table 4:** Instrumental conditions for both analytical methods used.

Parameter	ICP-OES ^1^	ICP-SFMS ^2^
Instrument	Prodigy High Dispersive ICP-AES (Teledyne Leeman, Hudson, NH, USA)	Element 2 ICP-SFMS (Thermo Fisher; Bremen, Germany)
Output power	1100 W	1300 W
Argon flows	Coolant:18 L min^−1^ Auxiliary: 0.8 L min^−1^ Nebuliser: 1 L min^−1^	Coolant:16 L min^−1^ Auxiliary: 0.86 L min^−1^ Nebuliser: 1.06 L min^−1^
Sample flow	1.0 mL min^−1^	100 μL min^−1^
Nebuliser	Pneumatic (glass concentric)	PFA microflow
Spray chamber	Glass cyclonic	PC ^3^ cyclonic quartz chamber
Plasma viewing	Axial	------

^1^ at Department of Chemistry, Faculty of Science, University of Zagreb. ^2^ at Division of Analytical Chemistry, Department of Chemistry, University of Natural Resources and Life Sciences Vienna.

## Data Availability

The data presented in this study are available on request from the corresponding author.

## Supplementary Materials

The following are available online, Table S1: Figures of merit of the analytical procedure.
