# Supplementary material for: Accumulation of Major, Minor and Trace Elements in Pine Needles (Pinus nigra) in Vienna (Austria)
Source: Molecules. 2021 Jun 1;26(11):3318. doi: 10.3390/molecules26113318 (PMC8198983; doi:10.3390/molecules26113318)
Supplement: Supplementary file 1 [file molecules-26-03318-s001.zip › molecules-1203350-SI.pdf]

Table S1. Figures of merit of the analytical procedure.

Element	LOQ [in mg/kg dry matter]	Precision [%]	Recovery* [%]
Silver	0.002	1.7	
Aluminium	1.9	2.6	99
Arsenic	0.02	1.1	91
Boron	0.32	2.7	91
Barium	0.01	0.7	95
Beryllium	0.001	1.5	
Calcium	0.42	1.9	101
Cadmium	0.006	1.4	98
Cobalt	0.004	0.8	100
Chromium	0.005	1.5	**
Copper	0.02	0.8	104
Iron	0.19	1.0	96
Potassium	0.84	2.4	95
Lithium	0.002	1.4	
Magnesium	0.61	1.4	96
Manganese	0.02	1.3	94
Molybdenum	0.002	1.5	
Sodium	0.20	2.3	107
Nickel	0.02	1.3	97
Lead	0.003	0.07	94
Selenium	0.02	2.6	107
Strontium	0.007	0.7	
Uranium	0.001	1.0	
Vanadium	0.003	1.4	
Zinc	0.04	0.7	99

*recovery only determined for the elements with certified or reference values for SRM 1575a. ** only range given for SRM.
